# Electron Acceleration to MeV Energies at Jupiter and Saturn

**DOI:** 10.1029/2018JA025665

**Published:** 2018-11-09

**Authors:** P. Kollmann, E. Roussos, C. Paranicas, E. E. Woodfield, B. H. Mauk, G. Clark, D. C. Smith, J. Vandegriff

**Affiliations:** ^1^ The Johns Hopkins University, Applied Physics Laboratory Laurel MD USA; ^2^ Max Planck Institute for Solar System Research Góttingen Germany; ^3^ British Antarctic Survey Cambridge UK

**Keywords:** electron radiation belt, adiabatic heating, wave acceleration, radial diffusion, Galileo/EPD, Cassini/MIMI

## Abstract

The radiation belts and magnetospheres of Jupiter and Saturn show significant intensities of relativistic electrons with energies up to tens of megaelectronvolts (MeV). To date, the question on how the electrons reach such high energies is not fully answered. This is largely due to the lack of high‐quality electron spectra in the MeV energy range that models could be fit to. We reprocess data throughout the Galileo orbiter mission in order to derive Jupiter's electron spectra up to tens of MeV. In the case of Saturn, the spectra from the Cassini orbiter are readily available and we provide a systematic analysis aiming to study their acceleration mechanisms. Our analysis focuses on the magnetospheres of these planets, at distances of L > 20 and L > 4 for Jupiter and Saturn, respectively, where electron intensities are not yet at radiation belt levels. We find no support that MeV electrons are dominantly accelerated by wave‐particle interactions in the magnetospheres of both planets at these distances. Instead, electron acceleration is consistent with adiabatic transport. While this is a common assumption, confirmation of this fact is important since many studies on sources, losses, and transport of energetic particles rely on it. Adiabatic heating can be driven through various radial transport mechanisms, for example, injections driven by the interchange instability or radial diffusion. We cannot distinguish these processes at Saturn with our technique. For Jupiter, we suggest that the dominating acceleration process is radial diffusion because injections are never observed at MeV energies.

## Key Point

- Electrons in Saturn's and Jupiter's magnetosphere, outside the most intense radiation belts, are accelerated through adiabatic transport

## 1 Introduction

The intrinsic magnetic fields of planets such as Jupiter, Saturn, and Earth are able to trap charged particles in the radiation belts encompassing them. The sources of these charged particles are, for example, rings, gas tori, solar wind, and planetary ionospheres. All these sources provide particles only at electronvolt (eV) and kiloelectronvolt (keV) energies. Yet the energies in radiation belts reach to megaelectronvolt (MeV) energies and above. How charged particles are accelerated to such high energies is not completely understood to date.

The problem of magnetospheric particle acceleration is reminiscent of the energization of galactic cosmic rays, which are also charged particles but at energies as high as 10^21^ eV (Swordy, 2001) that are not trapped in any magnetic field. Cosmic rays also originate at low energies and are accelerated to higher energies, for example, by encountering supernovae remnants (Achterberg, 1984). However, this acceleration can only be observed indirectly. The evolution of radiation belt populations, on the other hand, can be studied in situ and therefore provides a ground truth to the understanding of particle acceleration in general.

In planetary radiation belts and the magnetospheres encompassing them, there are several suggested processes that accelerate particles. Acceleration usually occurs in steps, where some processes accelerate to a certain energy and then other processes take over and provide acceleration to even higher energies. The dominant plasma source in the magnetospheres of Jupiter and Saturn is ionization of SO_2_ and H_2_O gas released from their moons Io and Enceladus, respectively (Gombosi et al., 2009; Thomas et al., 2004). Freshly produced ions and electrons start out with Keplerian energies on the order of eV but are picked up by the corotational electric fields of the planet that accelerates the particles roughly to the local corotation speed. Since electrons and ions have significant temperatures, the ratio of their peak energies is not determined by the electron/ion mass ratio. Instead, the energies where their spectra peak are comparable (Young et al., 2005). The plasma temperature may be a result of waves following the pickup process (Barbosa, 1986) and magnetohydrodynamic turbulence from reflected Alfvén waves (Saur, 2004), supported by thermal equilibration between ions and electrons (Rymer et al., 2007).

Radial transport through centrifugally driven interchange (Southwood & Kivelson, 1987) changes the energy of the particles. It moves corotating plasma with ≲1 keV outward. The change of corotation speed with distance yields heating with increasing distance. At the same time, interchange moves energetic particles inward, adiabatically increasing their energy with decreasing distance up to hundreds of keV (Rymer et al., 2008) but probably not higher than that since injections are not observed at ≥1 MeV. Another acceleration process results from the coupling of magnetosphere and ionosphere through electric currents (Cowley & Bunce, 2001; Hill, 1979) and their associated electric potentials near the poles (Knight, 1973; Saur et al., 2003). These potentials can reach ≈100 keV at Jupiter and are observed to accelerate electrons originating from the magnetosphere or ionosphere that follow magnetic field lines (Clark et al., 2017; Mauk et al., 2017).

What mechanism accelerates charged particles to even higher energies, beyond hundreds of keV to tens of MeV, is still under debate. A possible mechanism is interaction with whistler mode chorus waves. This mechanism is found to be important at Earth (Horne, Thorne, Shprits, et al., 2005; Miyoshi et al., 2003; Shprits et al., 2009; Thorne, 2010) and may also apply to the radiation belts of Jupiter and Saturn (Horne et al., 2008; Lorenzato et al., 2012; Nénon et al., 2017; Shprits et al., 2012; Woodfield et al., 2014). Another possibility is adiabatic heating through radial transport (Paranicas et al., 2010; Shprits et al., 2009). We will discuss these possibilities in sections 2.2 and 2.3.

Answering what processes are involved and which of them is dominating does not only answer a fundamental question in magnetospheric science but also provides a valuable tool for other studies: Particle distributions are commonly organized under the assumption of adiabatic heating (by calculating phase space densities at constant adiabatic invariants, see section 2.1). This then is the basis to study sources and sinks of these particles (Hood, 1983; Thomsen et al., 1977) or the transport in injections (Paranicas et al., 2016). However, while assuming that adiabatic heating is reasonable and models relying on that assumption are able to reproduce the data (Clark et al., 2014; Kollmann et al., 2017; Nénon et al., 2017), there were only few studies showing direct evidence for this: Dialynas et al. (2009) fit Saturn's energetic ion spectra for a temperature and found that it behaves adiabatically. Paranicas et al. (2010) found that the cutoff energy of the main energetic electron population during one Cassini orbit followed adiabatic heating.

In this paper we discuss in section 2 the mechanisms that can accelerate >100 keV particles to higher energies. The theory discussed in this section will guide us in the data analysis described in section 3. To test for wave acceleration, we will compare the data to model predictions (Woodfield et al., 2014). In order to test for adiabatic heating, we perform a similar study as Paranicas et al. (2010) but use all orbits of both Cassini and Galileo. Based on this combination of theory and data we will conclude in section 4 that the main acceleration process in giant planet magnetospheres, outside of their most intense radiation belts, is adiabatic transport.

## 2 Theory

### 2.1 General Adiabatic Heating

Charged particles trapped in a magnetic field undergo gyro, bounce, and drift motions (Walt, 1994). The drift around the planet roughly follows a circle on a constant *L* shell. We define the *L* shell here as the equatorial crossing point of a magnetic field line with the magnetic equator and will express it in multiples of a planetary equatorial radius. (Jupiter's equatorial radius is 1*R*_*J*_=71,492 km, Saturn's equatorial radius is *R*_*S*_=60,268 km.) This definition is a compromise between being straightforward to implement and realistically describing the drift. We discuss its properties in section 3.3.

An efficient way to accelerate a charged particle is by increasing the magnetic field it is exposed to. The field increase can happen when the particle is transported by some process to smaller *L*, closer to a magnetized planet. If the field increase is slower than the gyration and bounce time scale of the particle, acceleration occurs in an adiabatic way. Such an assumption is reasonable for transport processes as radial diffusion and interchange (Kellogg, 1959, section 2.2). If this assumption is indeed fulfilled, the first and second adiabatic invariants *μ* and *K*, associated with gyro and bounce motion, are conserved (Roederer, 1970).

(1) $$\mu =\frac{E\;(E+2mc^{2})}{2mc^{2}B}\sin^{2}\alpha =\frac{E\;(E+2mc^{2})}{2mc^{2}B}$$

(2) $$K=\overset{+\lambda_{m}}{\underset{-\lambda_{m}}{\int }}\sqrt{B_{m}-B}\;ds=0$$

*E* is the kinetic energy of the charged particle, *m* its rest mass, *c* the speed of light, *B* the local magnetic field, *B*_*m*_ the magnetic field at the mirror point (where the particle starts moving back to the equatorial plane), *α* the local pitch angle between the particle velocity *v* and the magnetic field, *λ* the magnetic latitude, and *λ*_*m*_ the latitude of the mirror point. The second equalities in each line of equation (1) are for equatorially mirroring particles with *α* = 90°, as we will assume it here for simplicity. Equation (1) shows that *E* increases with *B*. Equation (2) implies that the pitch angle remains at 90° for all *B* if *K* remains zero.

Nonequatorial particles (not used here) will slightly change their pitch angle with *B* and show a weaker change of *E*. The other extreme to an organized evolution of *α* following conservation of *μ* and *K* is that the pitch angle distributions efficiently isotropize at each *L*. Such a situation can be described by conserving an invariant proportional to the flux tube volume (Schulz, 1998; Toffoletto et al., 2003). Both the calculation of flux tube volume as well as of a nonzero *K* are sensitive to the magnetic field model and computationally expensive. In order to test if this would be worth the effort, we therefore start out with the simple case described above that relies solely on equatorially mirroring particles.

Approximating a planetary field with a dipole shows that the maximum change in energy through adiabatic heating depends only on the *L* shell range that can be traversed by a particle until other effects (wave acceleration, energy loss in neutral or plasma material) become important. The surface field strength of a planet plays no role in this approximation.

The abundance of energetic particles is commonly quantified by the differential intensity *j* (particles per time, area, solid angle, energy interval) or phase space density *f* (PSD, particles per volume in real and momentum space). They are related as

(3) $$f=\frac{j}{p^{2}}$$

with the momentum $p=\mathrm{mv}/\sqrt{1-v^{2}/c^{2}}$ and the total particle velocity *v*.

Radiation belt spectra can commonly (Garrett et al., 2012; Kollmann et al., 2011; Mauk et al., 2017; Paranicas et al., 2014; Figure 2b) be described to first order with a power law with exponent *γ* (not to be confused with the Lorentz factor used in relativistic calculations) followed by a cutoff or roll over at energy *E*_*c*_.

(4) $$j=p^{2}f={\left(\frac{E}{E_{0}}\right)}^{\gamma }\frac{j_{0}}{1+\exp\left((E-E_{c})/K_{T}\right)}$$

**Figure 1:**
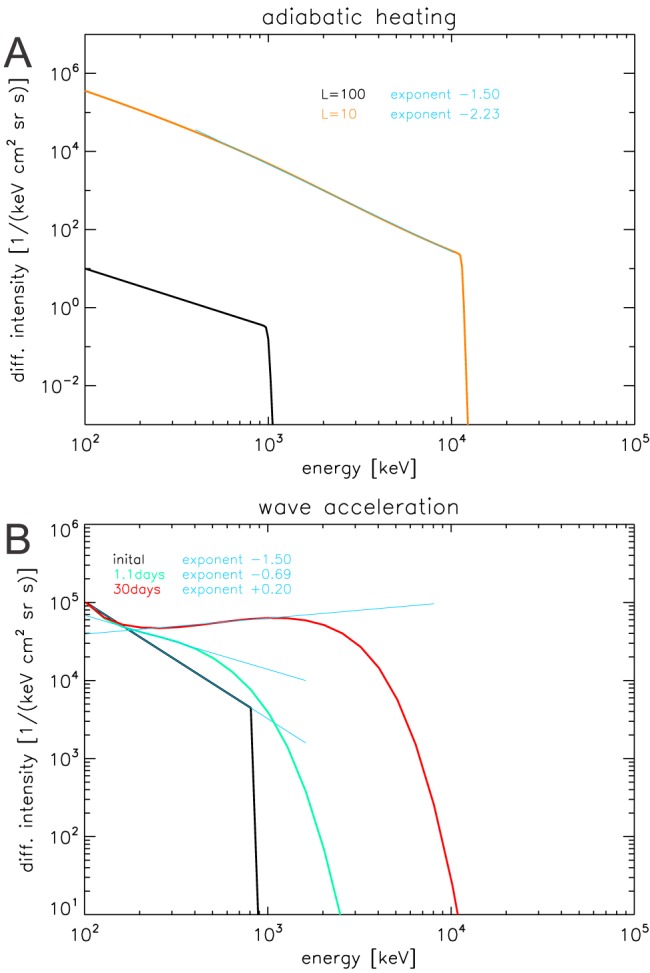
Illustration of the effects of different physical processes on the shape of electron spectra. (a) We assume that electrons start out at large distances to Jupiter with the power law spectrum shown in black. Adiabatic heating while moving closer to the planet shifts the spectra to higher energies (orange curve). While phase space density is conserved, the shown intensity increases due to equation (3). It can be seen that while the heated spectrum (orange) has a different shape and slope than the original spectrum (black), it still can be described reasonably well with a power law: The cyan curve shows a power law fit starting at E_0_=400 keV, the same energy we will use in section 3.1 for fitting the data. (b) We assume that, for example, an injection event quickly deposits the black spectrum in a region of significant energy diffusion. Energy (and pitch angle) diffusion first remove sharp gradients, yielding the spectrum shown in green. Energy diffusion then keeps accelerating electrons to higher energies (red). The spectra here show particles with α_eq_=86° and their time evolution is based on diffusion coefficients that are realistic for Jupiter at L = 10. The resulting spectra can be approximated by power laws (cyan curves), which illustrate that the power law exponent is increasing together with the cutoff energy, opposite to what we find for adiabatic heating (a).

*j*_0_ is the intensity at energy *E*_0_ and *K*_*T*_ determines how sharp the cutoff is. We show an example power law spectrum in Figure 1a assuming a sharp cutoff (*K*_*T*_=10 keV).

If all other processes (sources, sinks, acceleration, friction, etc.) are sufficiently slow, adiabatic heating conserves *f* (Schulz & Lanzerotti, 1974). Figure 1a illustrates that the spectrum after adiabatic transport does not follow a strict power law anymore. This PSD spectrum was constructed by shifting the *f* values at each *E* to higher energies, following equation (1). Since the relation between *μ* and *E* is nonlinear, each point in the spectrum is shifted in energy by another factor, which distorts the shape of the spectrum. The distorted spectra can still be approximated by a power law, however, with smaller *γ*, as also illustrated in Figure 1a. We will show the quantitative relation between the energy change and *γ* when we compare theory and data in Figure 6.

### 2.2 Adiabatic Heating Mechanisms

There are several mechanisms that can drive radial transport of charged particles into different magnetic fields, as it is required for adiabatic heating. For example, changes in a planet's corotational (Hill, 1979) or noon‐to‐midnight (Thomsen & Van Allen, 1980) electric field will change a particle's drift path and therefore *L* shell. Especially particles that are near‐stationary in local time because corotational and magnetic drifts (Thomsen & Van Allen, 1980) cancel out are sensitive to such changes (Roussos et al., 2018) and therefore efficiently heated. Such particles are MeV and tens of MeV electrons in the case of Saturn and Jupiter, respectively. Since Earth's magnetic field is directed opposite to Jupiter and Saturn, there are no local time stationary electrons at Earth. Earth therefore lacks one mechanism for efficient adiabatic transport.

**Figure 2:**
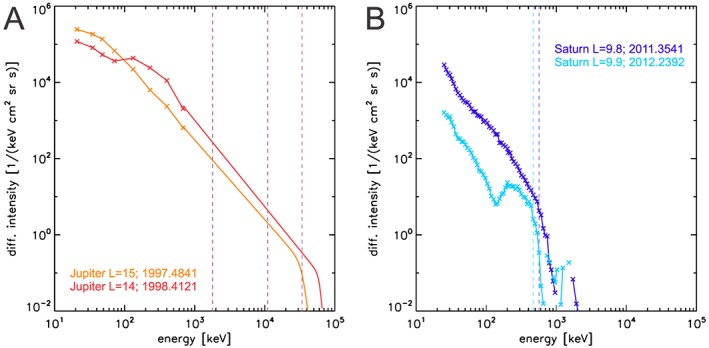
Typical electron spectra at Jupiter (a) and Saturn (b). For each planet we show one spectrum showing a dispersed injection (resulting in peaks at 100–200 keV) and one smooth spectrum. The Jupiter spectrum at <900 keV is based on differential intensity measurements. The center energies of the respective channels are marked with “x” symbols (these are E and F channels). At higher energies, spectra are based on a forward model (section 3.1) and measurements by three integral energy channels (B1, DC2, and DC3). The energies above which these channels measure with more than 50% of their maximum efficiency are marked by vertical lines. The energies of highest efficiency are higher than these thresholds (up to 80 MeV in the case of channel B1). For Saturn, we use the PHA_E and PHA_F channels. The vertical lines in the Saturn spectrum mark our automatically determined cutoff energies (section 3.2).

**Figure 3:**
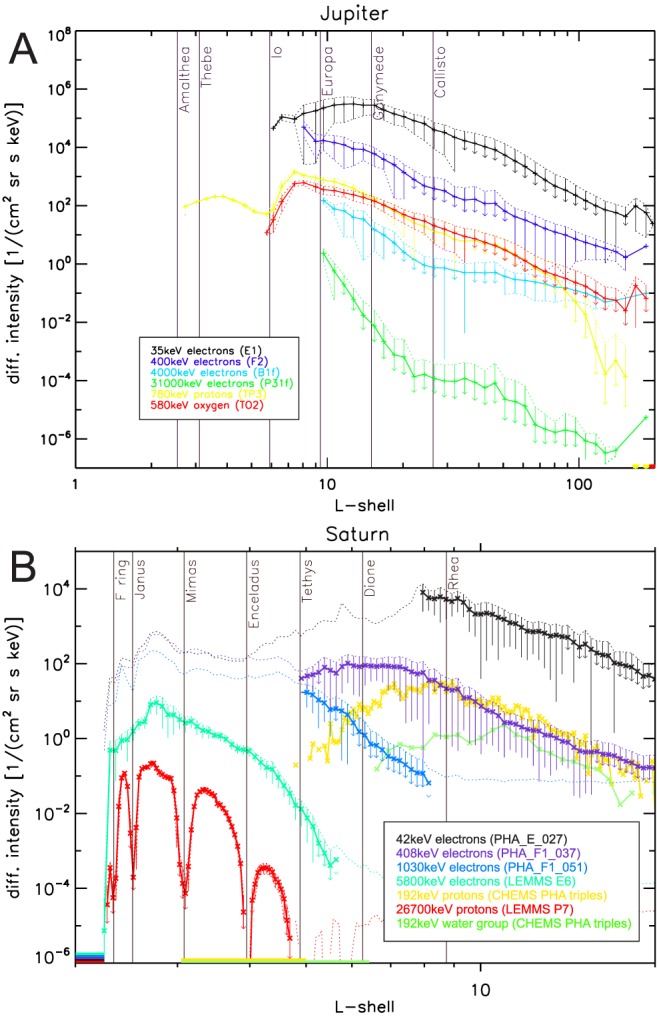
Overview of the radial intensity distributions around Jupiter from Galileo (A) and Saturn from Cassini (B). Jupiter data are omnidirectional for latitudes |λ|<15°, Saturn data are for equatorially mirroring particles with α_eq_≥30° and latitudes |λ|<10°. Thick curves show linearly averaged intensities after a median filter. Data where the measurements are unreliable are not shown in the case of Jupiter. At Saturn, we show unreliable intensities as dashed, thin curves that can still be used as upper limits. Colored bars on the lower horizontal axis indicate that valid data are present but zero on average. The legends provide the color code for particle species, center energy, and channel names. Error bars show the 1σ standard deviation, which usually indicates the true time variability of the intensities, not the Poisson uncertainty. The error bars are not shown for all species for clarity. If the lower end of a bar ends with an arrow, it indicates that it going to zero.

**Figure 4:**
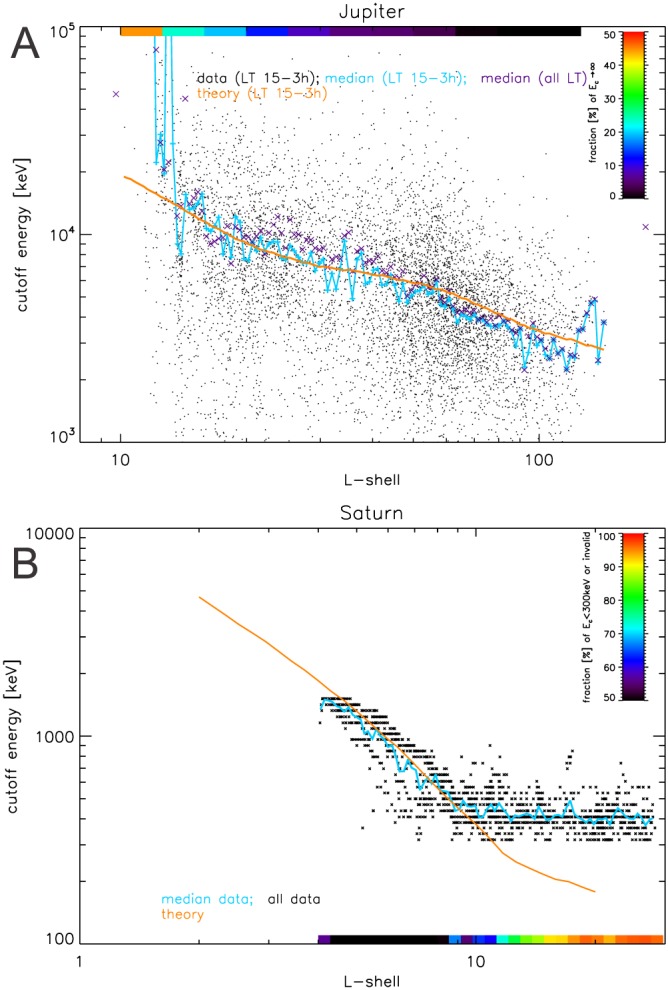
Evidence for adiabatic heating at Jupiter (a) and Saturn (b). Single points show all measurements throughout the missions. Their scatter illustrates that the shapes of the spectra are time variable. Jupiter data shown here is limited to the evening side with local times in the range 15–3 hr, Saturn data are limited to equatorial latitudes |λ|<10°. Cyan curves show the median cutoff energies, which can be directly compared to the expectation for adiabatic heating, shown in orange. To calculate adiabatic heating, we use the measured magnetic field strength and assume equatorially mirroring particles. It can be seen that the median data fit the adiabatic theory for wide L shell ranges. If the cutoff energy is above tens of MeV, their precise value cannot be determined with the available data. We still keep track of the occurrence of such high cutoffs and find that they make up a significant fraction of the Jupiter fits for L < 20, which is illustrated by the color coding on top of the Jupiter panel. Similarly, our method is unable to determine cutoffs at Saturn below 300 keV. We keep track of the fraction of measurements where we cannot determine their cutoff energy, which is illustrated by the color coding on the bottom of the Saturn panel. The median does not represent the cutoff energies well when a large fraction of values is invalid, as it is for L > 10 in the case of Saturn.

If the electric or magnetic field not only changes once but repeatedly, it randomly moves charged particles to different *L* shells relative to the planet. Such random changes to a particle population can be described by radial diffusion (Schulz & Lanzerotti, 1974; Walt, 1994). Field fluctuations may be driven by changes in the noon‐to‐midnight electric field (Andriopoulou et al., 2014), changes in corotation speed (Krupp et al., 2001), for example, due to variable mass loading from moons, the occurrence of injections and their associated field perturbations (section 2.2), turbulence in ionospheric winds and the resulting magnetospheric electric field (Brice & McDonough, 1973), or repeated compressions of the entire magnetosphere (Walt, 1994).

Observations support that radial diffusion is an important process at the giant planets. The most direct observation of radial diffusion is the gradual refilling of electron intensities in the drift shadow of moons (Roussos et al., 2007; Van Allen et al., 1980). The overall radial distribution of energetic particles is consistent with being shaped, along with other processes, by radial diffusion (Clark et al., 2014; Goertz et al., 1979; Hood, 1983; Kollmann et al., 2013; Lorenzato et al., 2012; Nénon et al., 2018; Santos‐Costa & Bolton, 2008; Thomsen et al., 1977). In the case of protons, not only their instantaneous distribution in space but also their year‐long intensity modulation over time is described well with radial diffusion (Kollmann et al., 2017).

Other mechanisms that can transport particles are dipolarization after tail reconnection (Jackman et al., 2007; Vasyliūnas, 1983) or centrifugally driven interchange (Chen et al., 2010; Southwood & Kivelson, 1987). Different to radial diffusion, injection and dipolarization only act in relatively narrow azimuthal ranges. In the swaths affected by these processes, the high PSD from large *L* shells replaces the smaller PSD at smaller *L* shells (Kidder et al., 2009; Liu & Hill, 2012; Paranicas et al., 2016). Such PSD enhancements are commonly called injections. The spectrum in an injection will change its shape adiabatically (section 2.1) only during ongoing radial transport. Then the injection disperses (Mauk et al., 2005; Müller et al., 2010) because particles of different energy have different drift speeds (Thomsen & Van Allen, 1980). After dispersion, the injection particles will be found at different azimuthal locations depending on particle energy. This results in peaked energy spectra, meaning that they cannot be described well with a power law anymore. An example for such a spectrum can, for example, be found in Figure 2b that is discussed below.

Injections at Jupiter and Saturn are only observed for energies <1 MeV (Clark et al., 2016; Paranicas et al., 2010). This is because the energy‐dependent gradient and curvature drifts cause high‐energy particles to leave the region with radial flow before crossing a significant radial distance (Burch et al., 2005; Paranicas et al., 2016). Injections are therefore very inefficient in transporting ≳1 MeV electrons. This means that we do not expect injections to play a dominant role in transporting or accelerating such high energies.

### 2.3 Wave‐Particle Interactions

A completely different acceleration mechanism is wave‐particle interactions with field fluctuations on the gyro timescale. Such fluctuations can, for example, result from whistler waves (Horne & Thorne, 2003). Particles randomly lose or gain energy, which can be described by energy diffusion (Schulz & Lanzerotti, 1974). Some recent measurements at Jupiter (Mauk et al., 2017) can be interpreted as showing neighboring regions of peaked initial spectra and broadened spectra after energy diffusion.

In order to illustrate how energy diffusion affects the spectral shape, we calculate the time evolution d*f*/d*t* of an initial PSD *f* (Glauert et al., 2014; Schulz & Lanzerotti, 1974; Walt, 1994).

(5) $$\frac{df}{dt}=\frac{1}{A}\frac{\partial }{\mathrm{\partial E}}\left(A\;D_{\mathrm{EE}}\frac{\mathrm{\partial f}}{\mathrm{\partial E}}\right)+\frac{1}{g}\frac{\partial }{\partial \alpha_{\text{eq}}}\left(g\;D_{\alpha \alpha }\frac{\mathrm{\partial f}}{\partial \alpha_{\text{eq}}}\right)$$

Since energy diffusion (first term in equation (5)) is highly pitch angle dependent, we also account for pitch angle diffusion (Elliott et al., 2018) through the second term in equation (5) but neglect cross diffusion. *D*_*EE*_ is the energy diffusion coefficient and *D*_*αα*_ the pitch angle diffusion coefficient. We use values for the diffusion coefficients as in Woodfield et al. (2014) that are realistic for Jupiter at *L* = 10.

The functions *A* and *g* are given by

(6) $$A=(E+E_{0})\sqrt{E(E+2E_{0})}$$

(7) $$g=\sin(2\alpha_{\text{eq}})\;T_{y}$$

(8) $$T_{y}=\frac{p}{4m_{e}LR_{p}}T_{B}\approx 1.3802-0.3198\left(\sin(\alpha_{\text{eq}})+\sqrt{\sin(\alpha_{\text{eq}})}\right)$$

with the electron rest energy *E*_0_, the electron rest mass *m*_*e*_, the planetary radius *R*_*p*_ (equals *R*_*J*_ or *R*_*S*_ depending on planet), the dimensionless *L* shell, and the bounce time *T*_*B*_. The approximation in equation (8) holds for a dipole magnetic field (Lenchek et al., 1961; Schulz & Lanzerotti, 1974), which works well for small *L* (section 3.3) and is therefore assumed here.

As initial conditions for equation (5) we assume a cutoff power law spectrum in energy (equation (4)) with parameters consistent with the observations (Figure 6). For the pitch angle distribution we assume $f\propto \sin(\alpha_{\text{eq}})$ for all energies, which is consistent with observations at small *L* (Clark et al., 2014; Tomás et al., 2004). As boundary conditions we set *f*(100 keV) being constant for all times and *f*(25 MeV) being negligible, similar to what was used in Woodfield et al. (2013) and Woodfield et al. (2014).

The time evolution of the spectra that results from energy and pitch angle diffusion is shown in Figure 1b. We fit the spectra assuming a power law spectral shape. (Since the low‐energy part of *f* is determined by the lower boundary condition, we fit only energies well above that lower boundary.) It can be seen that energy diffusion increases not only the cutoff energy but also the power law exponent. This behavior is opposite to what we found for adiabatic heating (compare the two panels in Figure 1). The qualitative correlation of the spectral parameters that results from energy diffusion is robustly found independent of the details of the calculation, particularly, it does not matter if different diffusion coefficients are used (Horne, Thorne, Glauert, et al., 2005; Horne et al., 2008), Earth instead of Jupiter is considered (Shprits et al., 2006), radial diffusion is included (Woodfield et al., 2014), or explicit pitch angle diffusion is neglected (Horne, Thorne, Glauert, et al., 2005).

### 2.4 Theory Summary

The acceleration mechanisms radial diffusion, injection, and energy diffusion show different correlations between the power law exponent *γ* and the cutoff energy *E*_*c*_. We summarize their behavior in Table 1.

**Table 1:** Summary of the Properties of Several Acceleration Processes

Process	Spectral shape	Adiabatic	Example
Radial diffusion	*γ* increases	yes	Jupiter ≥1 MeV,
	with increasing *E*_*c*_.		Figure 4a
	*E*_*c*_ increases		
	with time		
Injection	Not well described	yes	Saturn ≲1 MeV,
	by power laws		Figure 2b
Energy diffusion	*γ* decreases	no	Jupiter <1 MeV,
	with increasing *E*_*c*_.		Mauk et al. (2017)
	*E*_*c*_ increases		
	with decreasing *L*.		

*Note*. Injections can result from centrifugally driven interchange and dipolarization. Radial diffusion may be derived from a variety of processes, for example, changes in a planet's electric field (section 2.2). Radial diffusion and injections yield particle acceleration that is coupled with radial transport. Energy diffusion results from wave‐particle interactions. It yields local acceleration that is decoupled from radial transport.

The change in these spectral parameters happens as a function of *L* shell in the case of radial diffusion and injections and as a function of time in the case of energy diffusion. A single spacecraft cannot distinguish changes in space or in time and generally does not follow the radial transport of a particle or dwells at a site of local acceleration. It is therefore difficult to directly observe the ongoing evolution of an electron spectrum. What we are able to do instead is statistically study a large number of spectra. We will determine their *E*_*c*_ versus *L* correlation (to test for adiabatic heating, section 4.1) and their *E*_*c*_ versus *γ* correlation (to distinguish if adiabatic heating or energy diffusion dominates, section 4.2). The aggregate of all measurements will form tracks in *E*_*c*_ versus *L* and *E*_*c*_ versus *γ* diagrams. We suggest that single spectra pass along these tracks even though we cannot directly observe this. This approach is similar to using a Hertzsprung‐Russell diagram to understand stellar evolution that also needs to happen based on statistics since stellar lifetimes are too long to do this otherwise.

## 3 Measurements

### 3.1 Jupiter Radiation

In this work we use data taken around Jupiter and Saturn throughout large parts of the Galileo and Cassini missions. In the case of Jupiter, the data were taken by the Low Energy Magnetospheric Measurements System (LEMMS), part of the Energetic Particle Detector (EPD) suite on the Galileo orbiter (Hunt‐Ward & Armstrong, 2003; Mauk et al., 2004; Williams et al., 1992; and Appendix A). LEMMS consists of a high‐ and a low‐energy telescope (HET and LET, respectively). Both telescopes use stacks of solid state detectors (SSDs). The LET that measures <0.9‐MeV electrons separates these from ions through a magnetic deflection.

The Galileo mission was in orbit of Jupiter 1995–2003. This data set is unique even compared to contemporary data from the Juno mission since Galileo had an equatorial orbit. LEMMS was designed to provide measurements of >MeV electrons that are therefore relatively straightforward to calibrate. Due to the failure of Galileo's high gain antenna, the bulk of the data is available as omnidirectional intensities (Appendix A) with a typical time resolution of 10 min. This set is referred to as *real time* LGA‐0 data. While there are also data taken with higher time resolution, the advantage of the omnidirectional real‐time data is that they are available throughout most of the mission and have a large coverage in energies and particle species. The pitch angle distributions of electrons at the *L* shells and latitudes considered here are relatively flat (less than a factor of 10 over the full range, Tomás et al., 2004) compared to the dependence on energy (spectra cover several orders of magnitude, Figure 2). The omnidirectional measurements used here are therefore reasonably similar to the intensity at any other pitch angle.

Before our analysis, we first performed several corrections to the EPD data that are described in Appendix A.

LEMMS bins its counted particles into *channels* that cover various ranges of energy and particle mass. The channels for <0.9 MeV electrons measure relatively narrow energy ranges, which immediately allows to determine the differential intensity of these electrons. At higher energies, LEMMS only has channels measuring integral intensities above energy thresholds. In order to make use of these channels and derive differential intensities at MeV energies, we make use of a technique referred to as *forward modeling*. Our technique is similar to what was used for the GIRE model and its predecessors (Garrett et al., 2012; Jun et al., 2002). However, the GIRE standard products are intensities at a few energies and mission‐averaged spectra. This limitation made it difficult to test physical models (Horne et al., 2008; Woodfield et al., 2014) of electron acceleration at Jupiter. Therefore, we aim here to derive fully resolved spectra at all times.

The forward model relies on the relation between count rate *R*_*p*_ (counted particles per time) and differential intensity *j*

(9) $$R_{p}=\int_{0}^{\infty }dE\;j(E)\;G(E)$$

*G* is the geometry factor of the instrument that can be considered as the instrument's effective area and solid angle. We use values from Jun et al. (2002) based on their Monte Carlo radiation transport simulations of the instrument. (Note that the publication by Garrett et al. (2012) shows the same values as Jun et al. (2002) in their Figure 3 but listed outdated values in their Table 3.)

For the spectral shape we assume a power law with exponent *γ* followed by a sharp cutoff at energy *E*_*c*_ as in equation (4). We select *E*_0_=400 keV (the center energy of channel F2) and fix *K*_*T*_=3,000keV. The overall intensity *j*_0_ at *E*_0_ and the exponent *γ* are determined by fitting *j* from the differential energy channels F1, F2, and F3 that cover 174–884 keV (see Appendix A for detailed energy ranges of the EPD channels). The only free parameter remaining is the cutoff energy *E*_*c*_.

We apply the forward model to the channels B1, DC2, and DC3. Based on an initial guess of *E*_*c*_, we calculate predicted rates *R*_*p*_ based on equation (9). These rates are then compared with the actually measured rates *E*_*m*_ of these channels. We quantify the total error as $\Delta =\sqrt{\sum_{i}{\left(\log R_{p,i}-\log R_{m,i}\right)}^{2}}$. Usually, Δ is significant after the first iteration, so we change *E*_*c*_ until a match is found where Δ reaches a minimum. The change in parameters is done in an automatized way using the CONSTRAINED_MIN function available in the commercial software Interactive Data Language (IDL) by Harris Geospatial Solutions, Inc.

Example spectra based on the forward model are shown in Figure 2a. We perform forward modeling throughout the entire mission and find cutoff energies up to tens of MeV. An overview of electron intensities up to these energies is shown in Figure 3a. Mission‐average ion intensities are included for reference. We will discuss the spectral parameters accumulated over the mission in section 4.

**Figure 5:**
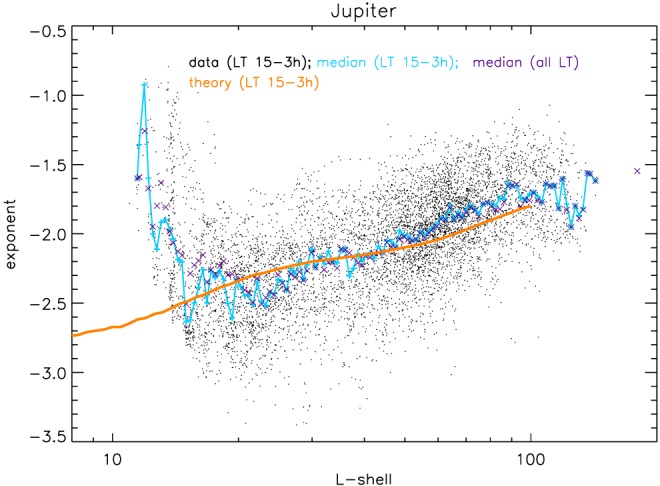
Further evidence for adiabatic heating at Jupiter. The figure shows measured power law exponents γ for electron spectra, compared to the expectation from adiabatic theory. Otherwise like Figure 4A.

### 3.2 Saturn Radiation

We use data taken throughout most of the Cassini mission. The instrument used is called LEMMS, as for Jupiter, and has a similar design to its Jupiter equivalent. In the case of Cassini, LEMMS is part of the Magnetosphere Imaging Instrument (MIMI) suite (Krimigis et al., 2004; Krupp et al., 2009).

We use the standard processing of LEMMS data (Vandegriff et al., 2013), and filter these data to measurements taken close to the magnetic equator (latitudes |*λ*|≤10^∘^) and away from the field‐aligned direction (only equatorial pitch angles 30^∘^≤*α*_eq_≤90° are kept). The data are then binned over 10 min to make them more statistically significant in low‐intensity regions. This also allows for better comparison with the Jupiter data that typically were also taken at this resolution. Since Saturn's proton radiation belts at *L* < 5 contaminate most of the LEMMS electron measurements in that region, we limit our analysis to the region outward of this.

As in the case of Jupiter, Saturn's electron spectra show a steep cutoff, even though it is at lower energies typically <1 MeV. We show example spectra in Figure 2b. The advantage of this low‐energy cutoff is that the MIMI/LEMMS instrument is able to measures this range with high‐energy resolution. Instead of integral channels as for EPD/LEMMS, we can use differential channels with ratios of energy range versus mean energy of 5–8% that is comparable to the standard products of contemporary radiation instruments. A forward model as in equation (9) is therefore not necessary to derive Saturn's electron spectra.

Disadvantage of a cutoff at low energies is that these energies are affected by injections, so that the spectrum near the cutoff generally cannot be described well with a simple power law. We therefore restrict our analysis on finding the cutoff energy for each measured spectrum. We determine the cutoff through an automatic procedure. Example results from this are shown as vertical lines in Figure 2b. We only use electron data with energies >200 keV using the instrument's F detector since the lower energies are covered by another detector that is not fully intercalibrated. Due to this restriction the lowest cutoff energy allowed in our fit procedure is 300 keV.

**Figure 6:**
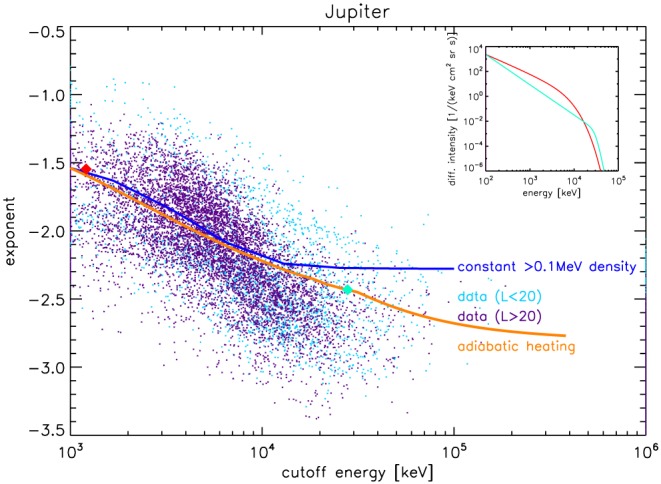
Correlation between spectral parameters of Jupiter's electron spectra. We show the power law exponent and the cutoff energy, at which the intensity is rapidly dropping (Equation (4)). The meaning of these parameters is illustrated through the inset: the red and green spectra in the inset have parameters highlighted by the red and green diamonds in the main figure. The correlation between the spectral parameters is in a way that the spectra become steeper (smaller exponent) when electrons are accelerated (higher cutoff). The measured behavior (small dots) is consistent with a redistribution of >0.1MeV electrons in energy while conserving their partial density n_p_ (blue line). It is also consistent with what we expect from adiabatic heating (orange line). Since Figures 4 and 5 suggest that adiabatic heating works best for L > 20, we show data inward and outward L = 20 in different colors (light and dark blue dots). This separation (that we also tested with smaller L‐bins) illustrates that the correlation is independent on L.

### 3.3 Magnetic Field

We organize the data through mapping the spacecraft location to the magnetic equator, where the total magnetic field reaches a minimum. We will refer to the distance of the crossing point to the center of the planet as *L* shell. Such mapping requires a magnetic field model. For Jupiter, we use the Khurana model (Khurana, 1997; Khurana & Schwarzl, 2005; Khurana et al., 2009) and for Saturn an offset dipole (Dougherty et al., 2005).

Magnetic field measurements are provided by the magnetometers of both missions (Dougherty et al., 2004; Kivelson et al., 1992). In the case of Cassini, we use the direction of the magnetic field to determine the local pitch angle between the particle velocity and the magnetic field. The used Galileo data are omnidirectional and do not require this.

For our analysis we also require the total strength of the magnetic field *B* in order to calculate adiabatic heating (equation (1)). Instead of using a magnetic field model for this, we filter the magnetic field measurements at both planets to magnetic latitudes |*λ*|≤10° and then average the field at each *L* shell. These averages are taken at the same locations as we will analyze the electron data.

Based on the average *B*(*L*) bins we find that Jupiter's field becomes nondipolar and shows an azimuthal asymmetry at L≳20. This implies that the *L* shell is not entirely conserved during the drift according to our *L* shell definition: Particles drifting at the largest measured distances (*L*≈100) can experience a change in *L* shell by a factor of <1.4. This imperfection is not an issue since the particle intensities at these distances are highly variable (Figure 3a), which does not change when considering only a narrow azimuthal range. In the case of Saturn the measured field becomes nondipolar at *L* > 10 but stays symmetric up to the largest distances of about *L* = 30 considered here.

## 4 Analysis

### 4.1 Test for Adiabatic Heating

The physical mechanisms that shape the electron spectra are expected to show different signatures in the data (section 2.4). At the current stage, we will not quantitatively consider a mix of several processes that might be necessary for an exact reproduction of the data. Instead, we will test if the data roughly follow the behavior expected from a single process, indicating that this process dominates over the others.

Our first test will be for adiabatic heating using the cutoff energy. Even though adiabatic heating shows in the entire spectrum, we will not study the full spectrum or test for conservation of PSD because other processes acting in parallel can also have an effect. Instead, we will track a strong spectral feature through different locations in the magnetosphere. We chose the spectral cutoff (equation (4)) as the tracked feature. This approach is robust against competing processes and easily applicable to a large data set.

If adiabatic heating has a major effect on the electrons, then the cutoff energy *E*_*c*_ will depend on *L* shell as described in equation (1). In Figure 4 we show the relation between *E*_*c*_ and *L* based on data and based on adiabatic theory. For both Jupiter and Saturn it can be seen that the median as well as the envelope of the measured cutoff energies systematically increase toward the planets.

In the case of Jupiter the increase in the cutoff energy toward the planet is generally consistent with adiabatic heating, at least for *L* > 20 (Figure 4a, curves). We find the best match when only considering spectra and measured magnetic fields taken on the evening hemisphere of the planet, for local times between 15 hr and 3 hr (with 12 hr being the sunward *noon* side and 24 hr being the *midnight* side of the planet), which roughly coincides with the hemisphere where the magnetic field lines are not strongly bent back in corotation direction (Khurana & Schwarzl, 2005) and where plasma sheet is thickest (Khurana et al., 2004).

The correlation of cutoff energy and *L* shell is suggesting that the dominant process accelerating >1 MeV electrons is adiabatic heating. While it is generally possible that a combination of nonadiabatic processes could mimic adiabatic behavior, it is unlikely that this would work out over such a large range of *L* shells as we observe it here. The driver for adiabatic heating needs to be radial diffusion from field fluctuations since injections from interchange or dipolarization are not observed for >1 MeV (Mauk et al., 1999; Paranicas et al., 2010). An alternative acceleration process would have been energy diffusion from interaction with whistler waves (Woodfield et al., 2014). Since the intensity of these waves is small for *L* > 20 (Menietti et al., 2016), it is maybe not surprising that acceleration occurs through adiabatic transport instead.

For *L* < 20, we find an increasing number of spectra with cutoff energies that are higher than what can be constrained with our Galileo data (Figure 4a, color scale). Particularly, these energies are higher than expected based on adiabatic theory, suggesting that it is not sufficient to explain electron heating in the radiation belts. We discuss this more in section 4.2.

For Saturn (Figure 4B), at least in the region of 4 < *L* < 10, we find that the observed change in cutoff energy is consistent with adiabatic heating. This suggests that Saturn's electrons are accelerated adiabatically in the magnetosphere despite the high intensity of whistler mode chorus waves around *L*≈6 (Menietti et al., 2014), which is consistent with theoretical predictions (Shprits et al., 2012).

The theoretical slope of *E*_*c*_(*L*) changes at *L*≈10 because the magnetic field is becoming less dipolar and therefore scales differently with *L*. Also, the measured slope of *E*_*c*_(*L*) changes around this location, but stronger than expected. However, this is mostly due to a bias in our data analysis. Measurements where the actual cutoff should be <300 keV have invalid *E*_*c*_ values in our data set, since 300 keV is the lowest cutoff energy we are able to determine with our current analysis (section 3.2). For *L* > 10 the fraction of invalid *E*_*c*_ reaches above 50% (Figure 4b, color scale), meaning that the average *E*_*c*_ value is not representative anymore.

The electron cutoff energy at Saturn is close to the resonance energy where the magnetic drift cancels out with the corotation drift (Thomsen & Van Allen, 1980), which makes these electrons quasi‐stationary in local time. Both observation and theory (Roussos et al., 2018) show that such stationary electrons are very efficiently transported by changes in the noon‐to‐midnight electric field (Thomsen et al., 2012). This changes the electron drift paths and may heat them adiabatically, depending on how fast the changes occur. Since changes in the electric field are at least common (Andriopoulou et al., 2014), maybe even continuous, their effect on electrons can potentially be described by radial diffusion.

### 4.2 Adiabatic Heating Versus Wave‐Particle Interactions

Since at Jupiter the power law exponent *γ* near the cutoff energy (>1 MeV) is unaffected by injections, *γ* can be used as a second test for adiabatic heating in the case of Jupiter, as discussed in section 2.4.

We show in Figure 6 that the two parameters describing the electron spectral shape, *γ*, and *E*_*c*_, are not independent but correlated: the higher the cutoff energy, the steeper the spectrum. This behavior is opposite to what we expect from wave acceleration (see Table 1) but consistent with our expectation for adiabatic heating (as illustrated by the overplotted theory curve). This indicates that adiabatic heating is the dominant process, at least in the region where most of our data were taken, at *L* > 20, outside of Jupiter's electron radiation belt. Wave‐particle interaction may be a second‐order effect at such large *L* shells. Its presence may explain the scatter and why the data do not perfectly fit adiabatic theory: Wave‐particle acceleration would locally (at every *L* shell it is active) act on the electron distribution to make the cutoff larger and the exponent less negative, as shown in Figure 1. In the statistical sense of Figure 6, this can result in a scatter of the points away from the adiabatic trend.

Figure 5 shows measured *γ* values as a function of *L* shell in comparison with the theoretical expectation. The behavior of *γ* is similar as of *E*_*c*_: adiabatic theory shows the same trend as the data for *L* > 20. Inward of this distance, the measured spectra are flatter than expected, suggesting that other processes contribute to shaping the electron spectra.

Whistler mode chorus wave intensities become significant for *L* < 20 (Menietti et al., 2016). (This is consistent with most injections observed in this region, Clark et al., 2016, which are associated with whistler mode waves, Bolton et al., 1997.) Based on the strong wave activity in this region, we suggest that wave acceleration may play a strong role within Jupiter's intense radiation belt at *L* < 20, which is consistent with theoretical predictions (Nénon et al., 2017; Shprits et al., 2012).

### 4.3 MeV Versus eV Acceleration

The *γ* versus *E*_*c*_ correlation shown in Figure 6 conserves the >0.1 MeV partial electron density *n*_*p*_. This density calculates as

(10) $$n_{p}=4\pi \overset{\infty }{\underset{p_{p}}{\int }}f\;dp$$

*p*_*p*_ is the momentum equivalent to *E*_*p*_=0.1MeV. The contour of constant *n*_*p*_ in Figure 6 shows that the data are consistent with conserving *n*_*p*_.

The full density *n*_*f*_ could formally be calculated by using *E*_*p*_=0. Calculating this in practice is difficult since such low energies are not measured by EPD. Such a calculation is not necessary though since the full density is dominated by thermal electrons, not the energetic electrons studied here (Bagenal & Delamere, 2011). This means *n*_*f*_ will be independent on the spectral parameters studied here. Contours of the full density are therefore horizontal lines in Figure 6 that do not fit the observed correlation.

The fact that the electron spectra are consistent with conserving *n*_*p*_ but not *n*_*f*_ suggests that whatever mechanism accelerates electrons to >10 MeV is only redistributing electrons that have >0.1 MeV, not *E*_*p*_≈0 eV. The MeV acceleration therefore does not feed directly from the reservoir of thermal eV electrons. Independent mechanisms (like pickup and injections) as they were discussed in section 1 need to accelerate the thermal electrons to ≈0.1 MeV first and by this provide the seed population. These mechanisms need to be independent on the acceleration to >10 MeV energies that are discussed here.

## 5 Open Questions

We find significant intensities of 1‐MeV electrons at Jupiter even at *L*≈100, very far from its intense radiation belts (Figure 4a). It is an open question where these electrons come from. From our data it is not clear what mechanism initially provides electrons with the observed spectra before they are transported inward and are energized further.

One possibility is that these electrons start as field‐aligned electrons that were accelerated at high latitudes by coherent electric fields. At Earth, such electric fields yield spectra with cutoffs similar as we observe here, even though their cutoff is with 1 keV at much lower energies (Carlson et al., 1998). The fields at Jupiter are stronger and reach at least 100 keV. Additional to the cutoff the Jupiter spectra show a sharp peak just at the cutoff energy (Mauk et al., 2017). This peak could not be resolved with the Galileo data and is likely diffusing away over time. Electron beams at Jupiter were also observed to reach to the MeV range (Paranicas et al., 2018), even though the driving mechanism is unclear. If any of these ≈100 keV to MeV electron beams map to distances large enough that the magnetic field becomes too weak or too curved, they may scatter in pitch angle, follow Speiser orbits (Speiser, 1965), remain at these large distances for some time, and by this provide the 1 MeV population visible in Figure 4a. This concept would be consistent with the observation electron pitch angle distributions being mostly field aligned at large *L* shells ( L≳17 for Jupiter, Tomás et al., 2004; L≳11 for Saturn, Clark et al., 2014).

Another theory that might explain the presence of MeV electrons at large distances is that they are derived from repeated adiabatic heating, where electrons run in a cycle of inward transport with heating at low latitudes and an energy‐preserving outward transport at high latitudes (Fujimoto & Nishida, 1990; Nishida, 1976).

## 6 Summary

1. Jupiter's ≳100‐keV electrons, at least in the low intensity, closed field line region of the magnetosphere at 20 < *L* < 100, are accelerated to >10‐MeV energies by adiabatic heating. This finding is supported by the change in cutoff energy and power law exponent with distance (Figures 4a and 5) and the correlation between these parameters (Figures 6).
2. Adiabatic heating at Jupiter likely occurs through radial diffusion since transport in injections would be inefficient at MeV energies.
3. For *L* < 20, toward Jupiter's intense electron radiation belt, the acceleration may involve wave‐particle interactions (section 4.2).
4. Jupiter's magnetosphere shows significant intensities of >MeV electrons even at distances of *L* = 100 from the planet (Figure 3).
5. Saturn's ≳100‐keV electrons at least in the range 4 < *L* < 10, outside the highest intensities of the electron radiation belt, are accelerated to >1 MeV energies by adiabatic heating. This finding is supported by the change in cutoff energy with distance (Figure 4b).
6. We suggest based on previous work (Roussos et al., 2018) that the driver for Saturn's adiabatic heating may be changes in Saturn's electric field (section 4.1).

## Figures and Tables

**Figure B1:**
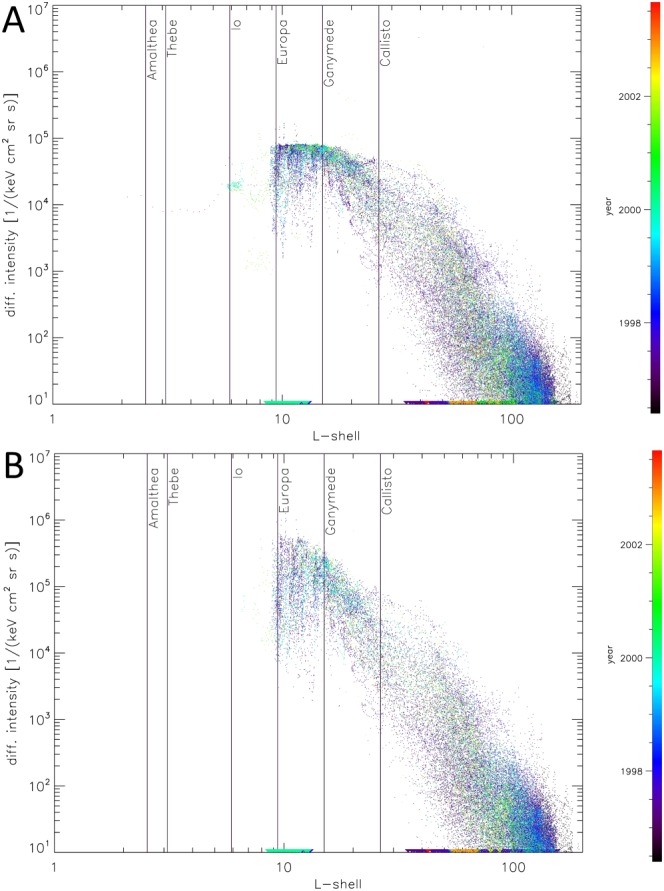
Intensity of 72‐keV electrons (E3 channel) throughout the mission before (a) and after (b) the corrections described in Appendix B. Most significant is the dead time correction that lets the uncorrected intensities of this channel saturate at ≈10^5^/(cm^2^·keV·sr·s), while the actual intensities can at times be a factor of ≈10 larger. This effect is most significant for data from the E detector measuring <93 keV electrons. The corrected data also removes most data of this energy inward of Europa's orbit since it is unreliable due to contamination. Some other channels provide reliable data up to smaller distances, see Figure 3a.

## Appendix A Galileo/EPD Channels

### A.1

EPD is an instrument suite (Hunt‐Ward & Armstrong, 2003; Williams et al., 1992) that includes the LEMMS and CMS instruments. Both are double ended and have a low‐energy and high‐energy telescope (LET and HET, respectively). CMS/LET is sometimes also referred to as CMS/TOFxE. CMS/HET consists of the two CMS/DeltaExE apertures that have each different geometry factors. Our work mostly uses data from LEMMS. Since not all information about EPD is publicly available, this appendix also documents some information about CMS.

The EPD instrument bins counted particles into *coincidence channels* that cover various ranges of particle energy and mass. The LEMMS coincidence channels use several SSDs in coincidence (triggering several detectors at the same time) or anticoincidence (triggering one but not another detector). *Singles channels* keep track of the total raw count rates of the SSDs. Downlinking the singles rates for detectors E and F was never planned and downlinking of channels As, EB2, and FB1 stopped in 1999. Since we require singles channels for some of the data processing, we reconstructed the singles channels by summing over the rates from the coincidence channels (A0, E0, etc.).

We provide a compilation of the calibration values for all Galileo/EPD channels in Tables A1 and A2. The upper and lower energies provided for the channels are usually their *nominal* passbands as they were already provided earlier (Lagg, 1998; Mauk et al., 2004; Williams et al., 1992). The center energies provided here are calculated as the logarithmic average of the passband boundaries, which is a standard approach. The center energy (sometimes also called mean energy) of a channel is ideally chosen as the energy where the fully resolved energy‐dependent intensity spectrum (if it were known with certainty) would equal the intensity averaged over the passband that is measured by the respective channel (Selesnick & Blake, 2000). This criterion is exactly fulfilled for the center energies used here if the energy spectrum is a power law with exponents of *γ* = 0 or *γ* =− 2. The geometry factors of the channels assume that intensity spectra are constant in energy, which is a common approximation. References to full response functions (geometry factor as a continuous function of energy) are provided in the comment column of the tables where available.

**Table A1:** Galileo/EPD/LEMMS Channel Parameters

		Geo. fact	Charge	Species	Low	Center	High						Footnote
Name	Instrument	(cm^2^ sr)			(keV)	(keV)	(keV)	Begin	End	HGA	LGA	Logic	(see Table A3)
A0	LEMMS/LET	0.0052	≥1	*M*≥1	22	30	42	1990 DOY350	1997 DOY305	64	6	A1 (A2 B1)	1
A0	LEMMS/LET	0.0052	≥1	*M*≥1	34	38	42	1997 DOY305	2004	64	6	A1 (A2 B1)	1
A1	LEMMS/LET	0.0056	≥1	*M*≥1	42	52	65	1988	2004	64	16	A2 (A3 B1)	1
A2	LEMMS/LET	0.006	≥1	*M*≥1	65	88	120	1988	2004	32	16	A3 (A4 B1)	1
A3	LEMMS/LET	0.006	≥1	*M*≥1	120	183	280	1988	2004	32	6	A4 (A5 B1)	1
A4	LEMMS/LET	0.006	≥1	*M*≥1	280	380	515	1988	2004	32	6	A5 (A6 B1)	1
A5	LEMMS/LET	0.006	≥1	*M*≥1	515	652	825	1988	2004	32	6	A6 (A7 B1)	1
A6	LEMMS/LET	0.006	≥1	*M*≥1	825	1,177	1,680	1988	2004	32	6	A7 (A8 B1)	1
A7	LEMMS/LET	0.006	≥1	*M*≥1	1,680	2,319	3,200	1988	2004	32	6	A8 (A9 B1)	1
A8	LEMMS/LET	0.006	≥2	*M*≥4	3,480	5,342	8,200	1988	2004	16	6	A9 (B1 C2)	2
E0	LEMMS/LET	0.015	−1	e	15	21	29	1988	2004	64	6	E11 (E12 E2)	3
E1	LEMMS/LET	0.038	−1	e	29	35	42	1988	2004	64	16	E12 (E13 E2)	3
E2	LEMMS/LET	0.04	−1	e	42	48	55	1988	2004	32	6	E13 (E14 E2)	3
E3	LEMMS/LET	0.038	−1	e	55	72	93	1988	2004	32	6	E14 (E15 E2)	3
F0	LEMMS/LET	0.025	−1	e	93	132	188	1988	2004	32	6	E15 (E16 E2); F11 (F12 F2)	3
F1	LEMMS/LET	0.017	−1	e	174	230	304	1988	2004	32	6	F12 (F13 F2)	3
F2	LEMMS/LET	0.016	−1	e	304	400	527	1988	2004	32	16	F13 (F14 F2)	3
F3	LEMMS/LET	0.012	−1	e	527	683	884	1988	2004	32	6	F14 (F15 F2)	3
B0	LEMMS/LET	0.0095	+1	H	3,500	5,916	10,000	1988	2004	16	1	A7 B1 (C2)	4; 4a
B1	LEMMS/LET	0.017	−1	e	36,200	—	∞	1988	2004	16	1	A2 (A4) B1 (B2 C2)	4
B2	LEMMS/LET	0.006	≥1	*M*≥4	16,000	40,000	100,000	1988	2004	16	1	A8 B3 (C2)	2; 4a
DC0	LEMMS/HET	0.005	±1	e+H	14,000	—	∞	1988	2004	16	1	(B1) D2 (C1)	4; 4a; 5
DC1	LEMMS/HET	0.005	±1	e+H	30,000	—	∞	1988	2004	16	1	(B1) C2 D1	4; 5
DC2	LEMMS/HET	0.580	−1	e	1,800	—	∞	1988	2004	16	1	(B1) D1 (D2)	4
DC3	LEMMS/HET	0.270	−1	e	10,800	—	∞	1988	2004	16	1	(B1) C1 (C2) D1	4
As	LEMMS/LET	0.006	all	all	22	—	∞	1988	1997 DOY305	16	1	A1	6
As	LEMMS/LET	0.006	all	all	34	—	∞	1997 DOY305	2004	16	1	A1	6
Bs	LEMMS/LET	0.006	all	all	>75	?	∞	1988	2004	16	1	B1	6; 7
Cs	LEMMS/HET	0.42	all	all	>162	?	∞	1988	2004	16	1	C1	6; 7
Ds	LEMMS/HET	0.42	all	all	>133	?	∞	1988	2004	16	1	D1	6; 7
EB1	LEMMS/LET	?	all	penetrators	?	—	∞	1988	2004	16	1	E16 (E2)	6
EB2	LEMMS/LET	?	all	penetrators	?	—	∞	1988	2004	16	1	E11 E2	6
FB1	LEMMS/LET	?	all	penetrators	?	—	∞	1988	2004	16	1	F15 (F2)	6
FB2	LEMMS/LET	?	all	penetrators	?	—	∞	1988	2004	16	1	F11 F2	6

*Note*. See Appendix A for column explanations.

**Table A2:** Galileo/EPD/CMS Channel Parameters

		Geo. fact			Low	Center	High						Footnote
Name	Instrument	(cm^2^ sr)	Charge	Species	(keV)	(keV)	(keV)	Begin	End	HGA	LGA	Logic	(see Table A3)
TP1	CMS/TOFxE	0.001	+1	H	130	169	220	1988	1996	16	16	START STOP KT	8; 10
TP1	CMS/TOFxE	0.001	+1	H	80	133	220	1996	2004	16	16	START STOP KT	8; 10
TP2	CMS/TOFxE	0.0011	+1	H	220	345	540	1988	2004	16	16	START STOP KT	9; 10
TP3	CMS/TOFxE	0.0006	+1	H	540	785	1,140	1988	2004	16	6	START STOP KT	10
TA1	CMS/TOFxE	0.0025	all	He	108	259	620	1988	2004	16	16	START STOP KT	10
TA2	CMS/TOFxE	0.003	all	He	620	1,575	4,000	1988	2004	16	6	START STOP KT	10
TO1	CMS/TOFxE	0.001	all	O + S	235	283	416	1996	2004	16	16	START STOP KT	11; 10
TO1	CMS/TOFxE	0.001	all	O + S	192	283	416	1996	2004	16	16	START STOP KT	12; 10
TO2	CMS/TOFxE	0.0025	all	O	416	583	816	1988	2004	16	16	START STOP KT	10
TO3	CMS/TOFxE	0.0035	all	O	816	1,209	1,792	1988	2004	16	16	START STOP KT	10
TO4	CMS/TOFxE	0.0035	all	O	1,792	4,014	8,992	1988	2004	16	6	START STOP KT	10
TS1	CMS/TOFxE	0.002	all	S	512	701	960	1988	2004	16	16	START STOP KT	10
TS2	CMS/TOFxE	0.003	all	S	960	1,380	1,984	1988	2004	16	16	START STOP KT	10
TS3	CMS/TOFxE	0.0035	all	S	1,984	4,436	9,920	1988	2004	16	6	START STOP KT	10
TH1	CMS/TOFxE	0.003	all	*M* ≫ 32	1,120	3,542	11,200	1988	2004	16	6	START STOP KT	13
TACs	CMS/TOFxE	0.007	all	all	?	—	∞	1988	2004	16	6	START STOP	6
KTs	CMS/TOFxE	?	all	all	?	—	∞	1988	2004	16	6	KT	6
STARTs	CMS/TOFxE	?	all	all	?	—	∞	1988	2004	16	16	START	6
CA1	CMS/DeltaExE	0.008	all	He	760	1,220	1,960	1988	2004	16	—	J (K)	14
CA3	CMS/DeltaExE	0.008	all	He	1,960	2,309	2,720	1988	2004	16	—	J K	14
CA4	CMS/DeltaExE	0.008	all	He	2,720	3,903	5,600	1988	2004	16	—	J K	14
CM1	CMS/DeltaExE	0.008	all	O	2,560	4,746	8,800	1988	2004	16	—	J (K)	14
CM3	CMS/DeltaExE	0.008	all	O	8,800	12,445	17,600	1988	2004	16	—	J K	14
CM4	CMS/DeltaExE	0.008	all	O	17,600	28,577	46,400	1988	2004	16	—	J K	14
CM5	CMS/DeltaExE	0.008	all	O	46,400	89,127	171,200	1988	2004	16	—	J K	14
CN0	CMS/DeltaExE	0.008	all	*M*≈23	23,000	34,115	50,600	1988	2004	16	—	J K	14
CN1	CMS/DeltaExE	0.008	all	*M*≈23	50,600	116,690	269,100	1988	2004	16	—	J K	14
CH1	CMS/DeltaExE	0.008	all	S	12,320	15,089	18,480	1988	2004	16	—	J (K)	14
CH3	CMS/DeltaExE	0.008	all	S	18,480	26,332	37,520	1988	2004	16	—	J K	14
CH4	CMS/DeltaExE	0.008	all	S	37,520	52,263	72,800	1988	2004	16	—	J K	14
CH5	CMS/DeltaExE	0.008	all	S	72,800	247,289	840,000	1988	2004	16	—	J K	14
Jas	CMS/DeltaExE/A	0.008	all	all	?	?	?	1988	2004	16	—	Ja	6
Jbs	CMS/DeltaExE/B	0.025	all	all	?	?	?	1988	2004	16	—	Jb	6
Ks	CMS/DeltaExE	0.034	all	all	?	?	?	1988	2004	16	—	K	6

*Note*. See Appendix A for column explanations.

**Table A3:** Footnotes to Tables A1 and A2

Number	Footnote
1	Table assumes protons but generally responds to all ions as provided in Mauk et al. (2004).
2	Table assumes helium but generally responds to all nonproton ions as provided in Mauk et al. (2004).
3	Values here are updated from Lagg (1998). Full response and effective geometry factors assuming nonflat spectra are shown in Lagg (1998).
4	Full response in Jun et al. (2002).
5	Table assumes electrons but also designed to respond to protons.
6	Only for diagnostic purposes.
7	Table energies are deposited in solid state detector.
8	Responds also to 3,870–16,000 keV protons.
9	Responds also to 1,140–3,870 keV protons.
10	Full response in Mauk et al. (2004) (use their Figure A1 or multiply their Table A2 with 0.533).
11	Table assumes oxygen but responds similar to 310‐512 keV sulfur.
12	Table assumes oxygen but responds similar to 253‐512 keV sulfur.
13	Contaminated.
14	Geometry factor was 0.034 cm^2^ sr before 1997 DOY 234.

For the B1 and DC channels, the lower passbands are defined here as the energy where the geometry factor (Jun et al., 2002) rises above half of its maximum. The geometry factor for these channels is the average value above this threshold. These values are only useful for zeroth‐order estimates of the intensity spectra. It is important to remember that channels usually also have a nonzero responsiveness outside of the passbands. Especially, channel B1 is already sensitive at MeV energies, well below its lower passband at tens of MeV. There are circumstances, for example, when the intensity at energies outside the channel passband is sufficiently high, that the total count rate may be dominated by energies outside of the passband, even if the channel is much more responsive to energies within its passband. A proper calculation of intensity spectra therefore should use the full response functions referred to in the table and use a technique similar to that described in section 3.1.

The species column in the tables describes the typical species measured by the channel. Sometimes this is expressed through the mass number *M* that is normalized to atomic mass units. Some channel definitions changed over the mission and we provide the time ranges. The abbreviation DOY means day of year and is counted from day 1 onward.

For each measurement interval, EPD bins measurements are taken at different directions into sectors. The more sectors, the higher the pitch angle resolution. The HGA and LGA columns in the tables refer to the number of sectors that were set up in record and real‐time mode, respectively. The LGA mode with one sector provides one omnidirectional measurement of the foreground, and additionally, there is a measurement of the background taken behind the calibration shield.

Most EPD channels use the measurements of several detectors in coincidence and/or anticoincidence in order to identify a particle. Each particle causes a pulse in the instrument electronics that is compared against various threshold values. The *logic* column in the tables explains the thresholds that need to be triggered in order for a particle to be counted by a given channel. The column entries provide the detector name (like A, E1, J, etc.) followed by the threshold number. Detector names J and K refer to both the detectors Ja and Ka, and Jb and Kb. A blank space toward the next entry means a logical AND. A semicolon means OR. An entry in brackets means that this detector/threshold is required to NOT trigger. For example, "E11 (E2)" means that threshold 1 of detector E1 needs to be triggered but that none of the thresholds of detector E2 are allowed to be triggered.

## Appendix B Galileo/EPD Data Processing

### B.1

A major issue of some of the Galileo/LEMMS data is that the instrument saturated when intensities became too high, which was usually the case close to Jupiter at *L* < 30. In these cases, the instrument provided high count rates that were only weakly dependent on the actual intensities. This behavior is illustrated in Figure B1: While intensities commonly show variations over up to 2 orders of magnitude, most intensities near the planet are at a constant level.

Saturation of measured count rates occurs because a particle instrument is after the detection of one particle unable to count the next particle before the passage of the instrument's dead time *T*_*D*_. If *N* particles arrive during a time Δ*T*, the rate *R*_*m*_=*N*/Δ*T* measured by a saturating instrument is related to the actual rate *R*_*a*_ as (Knoll, 2000)

(B1) $$R_{a}=\frac{N}{\Delta T^{\;u}N\;T_{D}}=\frac{R_{m}}{1-T_{D}R_{m}}$$

This equation is exact for the rates of singles channels (defined in Appendix A). It is approximately true for rates of channels using one detector with dead time *T*_*D*_ in anticoincidence with another detector (as it is the case for the E and F channels). For the channels that rely on the coincidence of two detectors, we use the detector with the higher singles rate in the saturation region (B detector for B channels, D detector for DC channels). For F0 that accepts counts from two detectors, we use the F detector, since its saturation behaves similarly to F0.

If the instrument saturates, the count rate becomes

(B2) $$R_{D}=\frac{1}{T_{D}}$$

irrespective of the actual rate of particle arrival (see, e.g., Knoll, 2000, section 4). Previous corrections to the LEMMS data mitigated the saturation by assuming dead times based on various calibration campaigns. However, this did not remove the shown behavior, since the correction is very sensitive on the assumed value of the dead time. In order to improve upon this, we determined the dead time based on in‐flight data by finding the maximum rate *R*_*D*_ of each SSD detector over the entire mission and used *R*_*D*_ and equation (B2) to calculate the dead time. For the detector “F” we find *R*_*D*_=9 × 10^5^/s and for all other SSD detectors *R*_*D*_=5 × 10^5^/s, all reasonably consistent with nominal dead times of *T*_*D*_≈1 μs.

Sample data after applying equation (B1) and the determined dead times are shown in Figure B1. It can be seen that our correction removes the saturation. The correction provides good estimates on the radial intensity distribution (Figure 3a) since the uncertainty in the dead time correction is less than the *L* dependence of the intensity. The procedure does not provide robust spectra around 130 keV, which is the transition energy between two different SSDs used in LEMMS (E and F detectors) that have different dead times. Since the spectrum around 130 keV is relatively flat, the results are sensitive to the dead time correction and even the corrected data cannot be used to reliably determine the spectral shape in this range. After applying the dead time correction, we therefore check if the corrected data show an intensity jump at energies transitioning between the E and F channels and remove the data from the E channel (that has a stronger correction) if that is the case. Also, to ensure that our analysis is not compromised by artifacts of the dead time correction, our forward model only relies on energies >174 keV (channels F1 and above).

The difficulty in analyzing data from energetic particle instruments is that their channels sometimes count particles outside of their nominal energy or species range. This behavior is often called contamination. In order to help in mitigating this issue, the LEMMS telescopes were mounted on a rotating platform that repeatedly placed the instrument behind a calibration shield. This shield was blocking the foreground intensities from the magnetosphere. Still, the instrument was usually showing nonzero rates even when blocking the foreground. In a low‐intensity environment counts measured in this orientation are mostly from a weak calibration source mounted in the shield. In the high‐intensity region close to Jupiter, the counts behind the shield are mostly from penetrating radiation. Since these penetrators are also present while the instrument does not point toward the shield, we subtract these background counts from the foreground counts. The HET does not fully go behind the calibration shield, which for LEMMS/HET is so significant that shielded and unshielded counts are always similar, even for particles that are expected to be stopped in the shield. We therefore do not subtract rates taken by any of the HET instruments (LEMMS/HET and CMS/DeltaExE). Since the background subtraction does not work perfectly, we remove data entirely if background and foreground become comparable within a factor of 4.

Based on the isotropic pitch angle distribution of channel B0, indicative of penetrators, we also removed data from channel B0 inward of *L* < 5.9. Event data reveal that also channels TP1, TO1, and TO2 are unreliable for *L* < 5.9 and that TH1 is always unreliable.
